# Seasonal dynamics of barbecue-derived PAH accumulation in recreational Nature Park soils: evidence from Bolu Gölcük, Türkiye

**DOI:** 10.1007/s10653-026-03047-5

**Published:** 2026-02-09

**Authors:** Melike Büşra Bayramoğlu Karşı, Ercan Berberler, Duran Karakaş

**Affiliations:** 1 Innovative Food Technologies Development Application and Research Centre, YENIGIDAM, Bolu Abant Izzet Baysal University, 14030 Bolu, Türkiye; 2 Department of Environmental Engineering, Bolu Abant Izzet Baysal University, 14030 Bolu, Türkiye; 3 Department of Environmental Engineering, Bartın University, 74100 Bartın, Türkiye

**Keywords:** Polycyclic aromatic hydrocarbons (PAHs), Spatial mapping, Seasonal variation, Barbecue emissions, Recreational soils

## Abstract

Polycyclic aromatic hydrocarbons are persistent and carcinogenic contaminants that accumulate in soils, posing ecological and public health concerns in recreational environments. This study examines the concentrations, ring-based composition, spatial distribution, and seasonal dynamics of 16 U.S. EPA priority PAHs in surface soils of Gölcük Nature Park (Bolu, Türkiye), a heavily visited recreational area where outdoor barbecuing is widespread. A total of 42 soil samples were collected during summer and winter 2016, and PAH concentrations were quantified using a rigorously validated high-performance liquid chromatography (HPLC) method incorporating matrix-matched calibration and procedural blank correction. ΣPAH_16_ spanned 108.3–2587.8 ng/g dry weight (dw) in summer and 111.8–3125.8 ng/g dw in winter, with higher winter levels reflecting cumulative atmospheric deposition following intense late-summer and early-autumn recreational activities. Molecular-weight- and ring-based assessments revealed the consistent dominance of high-molecular-weight PAHs, particularly 5–6 ring species, driven by their low volatility and strong sorption. Spatial interpolation maps generated in MapInfo (IDW) identified pronounced seasonal shifts in ΣPAH_16_ and HMW PAH hotspots, especially in the eastern lakeside picnic–barbecue zone, whereas shaded forested areas exhibited distinct photo-oxidative attenuation patterns. According to the Maliszewska-Kordybach classification, up to one-third of winter samples corresponded to heavily contaminated soils. Benchmarking against a reference lake and international datasets indicates that PAH levels in this protected nature park exceed those of many urban green spaces and approach concentrations typical of industrial settings. Overall, the findings demonstrate that recreation-derived emissions substantially degrade soil quality and highlight the need for evidence-based management strategies to prevent long-term ecological deterioration in protected natural areas.

**Supplementary Information:**

The online version contains supplementary material available at 10.1007/s10653-026-03047-5.

## Introduction

Parks, whether featuring naturally occurring or artificially planted vegetation, walking paths, and ponds, are among the most actively functioning areas within urban environments (Bakhmatova et al., 2022). Urban green spaces represent ecosystems closest to natural systems and play a critical role in preserving the urban ecological environment while supporting the physical and mental well-being of city residents (Qu et al., 2020). Outdoor barbecuing, particularly common in recreational areas, is one of the most widespread cooking activities worldwide and, although not performed daily, constitutes a significant source of ambient air pollution with potential health risks (Figueiredo et al., 2024). The partial combustion of organic materials during such activities can release substantial amounts of pollutants, including polycyclic aromatic hydrocarbons (PAHs) (Lao et al., 2018). PAHs are typical persistent organic pollutants (POPs) characterized by widespread occurrence, toxicity, carcinogenicity, and high environmental stability, and include sixteen compounds listed as priority pollutants by the U.S. Environmental Protection Agency (USEPA) (Karaca, 2016; USEPA, 1993; Zhang et al., 2013). Due to their toxicity, carcinogenic potential, and environmental persistence, PAHs represent a significant ecological and public health concern, particularly in recreational areas where activities such as outdoor barbecuing can result in measurable soil contamination. Since urban soils can adversely affect human health due to the accumulation and release of pollutants such as PAHs, investigating these impacts—particularly in urban green spaces—is critically important from an environmental protection perspective.

To provide context for the 2016 soil sampling conducted in this study, previous research investigating PAH contamination in soils both in Turkey and internationally is summarized. In Turkey, reported PAH concentrations in urban and industrial soils and sediments vary widely, reflecting local anthropogenic pressures: for instance, in Istanbul botanical gardens, 2–3 ring, 4–6 ring, and 7-ring PAHs ranged approximately 50–80 μg/kg dry weight (Güner, 2009), while sediments from industrialized regions of the Marmara Sea reached 825–2154 ppm (Demircioğlu, 2011). Soils from multiple urban and rural sites in Istanbul showed mean concentrations of 21.76 ng/g dw for sixteen PAH compounds (Kıstaubayeva, 2015). Internationally, total PAH concentrations also display broad variation: in Tianjin, China, bulk and root-associated soils ranged 1–8 µg/g (Tao et al., 2004); in Norway, southern soils showed approximately tenfold higher 4–5 ring PAH levels compared to central regions due to long-range transport (Aamot et al., 1996); in Estonia, surface soils from areas with differing anthropogenic influence contained 11–153,000 mg/kg organic carbon, with site averages from 2,240 to 12,390 mg/kg OC (Trapido, 1999). These studies collectively highlight how local and regional human activities, as well as environmental transport processes, affect PAH accumulation in soils.

This study was conducted in Bolu Gölcük Nature Park, one of the most important recreational areas in Türkiye’s Western Black Sea and Eastern Marmara regions, due to long-standing and increasing visitor pressure as well as recent ecological degradations observed in the park (e.g., dieback in certain tree species and a marked decrease in lake water depth). Within the scope of the study, 21 surface soil samples were collected from around the lake, and the sixteen priority PAH compounds identified by the USEPA were analyzed during both summer and winter seasons. The primary aim of the research was to identify PAH pollution originating from barbecue activities, determine PAH concentration levels entering the soil through atmospheric deposition, and reveal the seasonal variation of this contamination.

The findings demonstrated that the surface soils of Gölcük Nature Park exhibited higher PAH accumulation levels than many industrial-region soils reported in the literature (Çetin, 2016; Hussain & Hoque, 2015; Kwon & Choi, 2014; Peng et al., 2011). This indicates that intensive recreational activities can lead to PAH accumulation in protected areas at levels comparable to those observed in industrial environments. Therefore, this study represents one of the pioneering investigations in Türkiye examining recreation-derived PAH pollution in protected natural areas, filling a significant gap in the literature and providing the scientific basis necessary for developing sustainable management strategies.

## Materials and methods

### Sampling area

The research was conducted within Bolu Gölcük Nature Park, a well-known protected area in Türkiye that has served as a popular recreational destination for nearly six decades. The park, located approximately 13 km south of Bolu city center (coordinates: 40°65′57″ N, 31°62′69″ E), encompasses a small, shallow lake with a surface area of about 45,000 m^2^, an average depth of 5.5 m, and a shoreline length of approximately 1300 m. The lake is encircled by dense fir (pine) forests, contributing to the park’s characteristic landscape. Within the park boundaries, there are four public restrooms, two restaurants, a mosque, two playgrounds, four bungalow-type lodgings, and designated picnic and barbecue sites. Motor vehicles are not permitted beyond the park entrance; a parking lot is situated roughly 50 m downwind of the lake. The remaining three sides of the lake are bordered by mountainous and hilly terrain, which limits air circulation and often leads to stagnant atmospheric conditions. The dominant wind direction in the area typically flows from west–west southwest (W–WSW) toward the park entrance north–northeast (N–NE) (Bayramoğlu Karşı et al., 2019). The park receives more than 350,000 visitors annually, with barbecuing representing the primary source of local atmospheric emissions, alongside recreational activities such as hiking, cycling, and amateur fishing.

### Reagents

All chemicals utilized in this study were of analytical grade or higher purity. N-hexane, methanol, dichloromethane (DCM), dimethylformamide (DMF), acetonitrile (ACN), tetrahydrofuran (THF), silica gel (pore size 60 Å, 70–230 mesh), and anhydrous sodium sulfate were supplied by Sigma-Aldrich (St. Louis, MO, USA). A PAH-mix-9 standard solution containing sixteen priority PAHs at concentrations of 10 ng/µL per compound—including naphthalene (NaP), acenaphthylene (Ace), acenaphthene (Acy), fluorene (Flr), phenanthrene (Phe), anthracene (Ant), fluoranthene (Flu), pyrene (Py), benz(a)anthracene (BaA), chrysene (Chr), benzo(b)fluoranthene (BbF), benzo(k)fluoranthene (BkF), benzo(a)pyrene (BaP), dibenz(a,h)anthracene (DahA), benzo(g,h,i)perylene (BghiP), and indeno(1,2,3-cd)pyrene (IcP)—was obtained from Dr. Ehrenstorfer GmbH (Augsburg, Germany). All stock and working standard solutions were stored in amber glass vials at − 20 °C to prevent photodegradation and volatilization. Prior to use, all glassware was thoroughly cleaned with hot detergent solution, subsequently rinsed with n-hexane, ultrapure water, and acetone, and then oven-dried at 110 °C to eliminate potential contamination.

### Sample collection

**Fig. 1:**
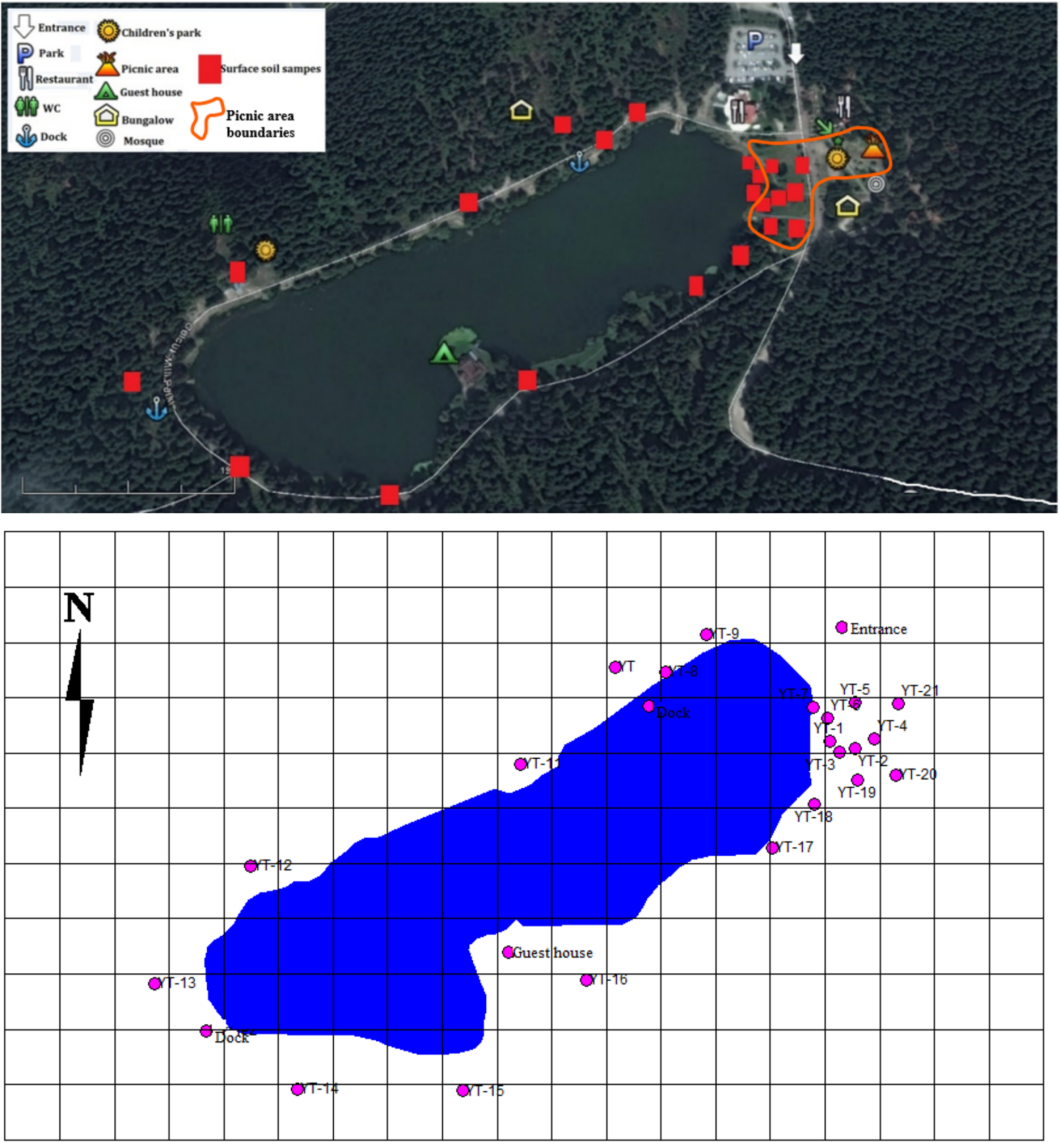
Sampling area and sampling points

Surface soil samples were collected from a depth of 0–10 cm using a stainless-steel shovel, with approximately 100 g of soil obtained per sampling point. The freshly collected samples were wrapped in aluminum foil, sealed in polyethylene zip-lock bags, labeled. All samples were transported to the laboratory on the same day. In the laboratory, plant roots, stones, and other foreign materials were carefully removed. The soil samples were then spread on aluminum foil and oven-dried at 35 °C overnight to remove moisture. Subsequently, the dried samples were ground using a mortar and pestle, and moisture content was determined gravimetrically using the conventional oven-drying method at 105 °C. Surface soil samples were collected twice during the same year—once in June (summer sampling) and once in November (winter sampling)—from 21 different sampling locations (Fig. 1). During the summer campaign, the GPS coordinates of all sampling sites were recorded to ensure that the same locations were revisited for the winter sampling. Gölcük Nature Park, where the study area is located, is characterized by a high elevation and harsh continental climatic conditions, resulting in frozen ground and dense snow cover for a substantial part of the year. From mid-November onward, freezing of the lake surface and increasing snow thickness render access to the soil surface technically unfeasible during the winter period. Therefore, winter sampling was conducted in November, immediately before the onset of freezing and snowfall. Summer sampling was carried out in June, when recreational activities and barbecue use within the park intensify. This sampling strategy allowed two campaigns per year representing periods of high activity (summer) and low activity (pre-winter) within the annual cycle. In addition, limitations in project budget and increasing fieldwork costs prevented the implementation of more frequent sampling campaigns. Sampling points were selected to represent different land-use intensities within the park. A higher density of sampling locations was established within the designated picnic area where barbecuing is permitted, while additional points were selected at regular intervals along the walking path surrounding the lake. At each sampling point, a single surface soil sample was collected from the 0–10 cm depth. The study was designed as an exploratory (screening-type) investigation aimed at comparing PAH accumulation levels among different functional areas of the park rather than quantitatively characterizing micro-scale spatial heterogeneity within individual sites. One week before the summer sampling, 10.4 mm of rainfall was recorded, with an additional 2.4 mm occurring on the day prior to sampling. The average ambient temperature during the week preceding the summer sampling was approximately 19 °C. Prior to the winter sampling, 0.2 mm of precipitation was recorded within two days before sampling, and the average temperature during the preceding week was 10.8 °C. Temperature and precipitation data were obtained from the meteorological station of Bolu Province to represent the sampling periods. The data corresponding to 1 May–30 June 2016 and 1 October–30 November 2016 are presented in Supplementary Figs. S1 and S2. Additionally, within the boundaries of Bolu province, three surface soil samples were collected from the vicinity of a lake located in an area distant from settlements, major roads, and direct or indirect pollution sources, and PAH analyses were conducted on these samples. The soils from this lake area were used as reference samples for comparison with the soil samples collected from Gölcük Nature Park. To minimize the influence of elevation differences on PAH concentrations, the elevation of the reference lake was carefully selected. The elevation of the reference lake is 1217 m, while that of Gölcük Nature Park is 1212 m.

### Extraction and clean-up

Approximately 25 g of soil was transferred into capped centrifuge tubes, followed by the addition of 20 mL of tetrahydrofuran (THF). The samples were vortexed for 2 min to ensure homogenization and then shaken in a water bath (Wise Stir WSB-18, DAIHAN Scientific) at 40 °C and 150 rpm for 2 h. After extraction, they were centrifuged at 4000 rpm for 6 min (ISOLAB Laborgeräte GmbH), and the supernatant was collected. The solvent was evaporated under a nitrogen stream to a final volume of 1–3 mL. For purification, a column containing pre-cleaned glass wool, 2 g of silica gel, and 1 g of anhydrous Na_2_SO_4_ was prepared and conditioned with 15 mL of n-hexane and 15 mL of dichloromethane (DCM). The extract was loaded onto the column, eluted with 4 mL of n-hexane and 6 mL of n-hexane/DCM (1:1, v/v), and the PAH fraction was collected. The eluate was concentrated to 1–2 mL under nitrogen, transferred into amber vials, and 50 μL of dimethylformamide (DMF) was added. After vortexing for 30 s, the samples were evaporated to near dryness (≈50 μL) and diluted to 1 mL with acetonitrile. The final extracts were vortexed again and stored at + 4 °C until analysis (Karşı, 2025).

### Sample analysis and instrumentation

PAH quantification was carried out using an Agilent 1100 Series high-performance liquid chromatography (HPLC) system equipped with a diode array detector (DAD). Separation of individual PAH compounds was performed on a Zorbax Eclipse analytical column (4.6 × 250 mm, 5 μm particle size). The mobile phase consisted of a water–acetonitrile mixture operated under a gradient program of 60:40 (0–0.66 min), 0:100 (0.66–36 min), and 60:40 (36–40 min) at a constant flow rate of 2.0 mL min⁻^1^. The detection wavelength was set to 220 nm, and the injection volume was 20 μL (Bayramoğlu Karşı et al., 2019).

### Quality assurance and quality control

For PAH quantification, calibration standards (0–3000 ppb, μg/L) were prepared using a representative matrix obtained by combining 200 μL aliquots of pre-extracted samples. Calibration was performed via the standard addition method, with the unspiked matrix serving as the blank. The blank signal was subtracted from the spiked standards to minimize matrix effects, improving analytical accuracy and reducing retention time shifts. Method detection limits (MDL) and limits of quantification (LOQ) were calculated from a 50 ppb spiked standard measured ten times, with the standard deviation multiplied by 3 and 10, respectively, and values applied to the calibration equation. Recovery tests were conducted using NIST SRM 1647d, diluted 20- and 50-fold, spiked into three samples, and processed following the same procedure (Pekey et al., 2004). Compound-specific MDL and LOQ values, together with analytical precision expressed as %RSD based on replicate analyses, are summarized in Table 2.

### Isomer ratios

For all samples, the sources of PAH compounds were identified using diagnostic ratios found in the literature, including Flr/(Flr+Py),BaA/(BaA+Chr), IcP/(IcP+BghiP), BaP/BghiP, Flu/(Flu+Py), and ΣLPAH/ΣHPAH (where LPAH refers to NaP+Ace+Acy+Flr+Phe+Ant and HPAH refers to Flu+Py+BaA+Chr+BbF+BkF+BaP+BghiP+IcP), Diagnostic ratios (DR) were calculated for each individual sample using concentrations of individual PAH compounds. Seasonal source characteristics were evaluated by calculating diagnostic ratios separately for the summer and winter datasets. Source contributions were interpreted based on threshold values reported in the literature (Akyüz & Çubuk, 2010; Katsoyiannis et al., 2007; Ravindra et al., 2008; De La Torre-Roche et al., 2009; Yunker et al., 2002; Zhang et al., 2008) (Table 1).

**Table 1:** PAH diagnostic ratios

Diagnostic ratio	Source	References
Flr/(Flr+Py)	> 0,5 Diesel combustion	Ravindra et al., (2008)
	< 0,5 Gasoline combustion	
Flu/(Flu+Py)	0,4–0,5 Fossil fuel combustion	Roche et al., (2009)
	> 0,5 Grass, wood, coal combustion	
	0,2–0,35 Coal combustion	
BaA/(BaA+Chr)	> 0,35 Vehicle emissions	Akyüz & Çubuk (2010)
	< 0,2 petrogenic	
	> 0,35 High temperature combustion	
IcP/(IcP+BghiP)	< Petrogenic	Yunker et al., (2002)
	0,2–0,5 Petrouem combustion	
	> 0,5 Grass, wood, coal combustion	
BaP/BghiP	< 0,6 non-Traffic emissions	Katsoyiannis et al., (2007)
	> 0,6 Traffic emissions	
LPAH/HPAH	< 0,1 Pyrogenic	Zhang et al., (2008)
	> 1,0 Petrogenic	
	~ 1,0 High temperature combustion	

The applicability of multivariate receptor models (principle component analysis and positive matrix factorization) was evaluated; however, these methods were not applied due to extensive data gaps and a high proportion of values below detection limits. Therefore, source apportionment was conducted using DR analysis.

### Statistical analysis

Statgraphics Centurion XV V. 15.2.11 was used to perform the statistical analysis. Normality of individual PAH concentrations for summer and winter datasets was evaluated based on standardized skewness and kurtosis values. Since a substantial number of variables did not satisfy the normality assumption, non-parametric statistical tests were applied in subsequent analyses. Differences among individual PAH concentrations within each season were evaluated using the Kruskal–Wallis test. Seasonal differences in total PAH concentrations (Σ16 PAHs) were assessed using a two-sample Kolmogorov–Smirnov test. Pearson correlation analysis was performed to examine relationships among individual PAHs. All statistical analyses were conducted at a 95% confidence level (*p* < 0.05). MapInfo Professional 9.5 was used for graphical illustrations.

### Risk assessments

Incremental Lifetime Cancer Risk (ILCR), an indicator of the increased likelihood of developing cancer due to exposure to potentially carcinogenic substances, was applied in this study to quantitatively assess the exposure risk associated with environmental PAHs, following the standardized frameworks established by the USEPA (2006).

The study population was stratified by age and sex, resulting in six exposure groups: children (2–15 years), adolescents (15–24 years), and adults (24–74 years), with each age category further divided into male and female subgroups.

To evaluate the carcinogenic potential of PAHs, concentrations of benzo[a]pyrene (BaP) and toxicity equivalent concentrations (TEQs) were calculated using the toxic equivalency approach:

1 $${\mathrm{TEQs}} = \sum {\mathrm{C}}_{{\mathrm{i}}} \times {\mathrm{TEF}}_{{\mathrm{i}}}$$

where C_i_ represents the concentration of the ith target PAH compound, and TEF_i_ is the corresponding toxic equivalency factor. The TEF for BaP was set to 1, according to established literature (Yu et al., 2008).

Human exposure to BaP in soil environments may occur through multiple pathways. These include (1) ingestion of BaP accumulated in plants, (2) inhalation of BaP associated with airborne particles, and (3) combined ingestion, dermal contact, and inhalation exposure resulting from direct interaction with contaminated soils.

In ILCR calculations, Cs represents soil PAH concentrations (mg/kg as TEQs), and CSF denotes the carcinogenic slope factor for each route (mg/kg·day). Other parameters include body weight (BW, kg), lifetime (AT, days), exposure frequency (EF, days/year), exposure duration (ED, years), inhalation rate (IRair, m^3^/day), soil ingestion rate (IRsoil, mg/day), exposed skin surface area (SA, cm^2^), soil adherence factor (AF, mg/cm^2^), dermal absorption fraction (ABS), and particulate emission factor (PEF, m^3^/day). A detailed description of all parameters is provided in Table S3 (Bayramoğlu Karşı, 2025).

The Incremental Lifetime Cancer Risk associated with soil ingestion, dermal contact, and inhalation pathways was calculated using the following equations:

2 $${\mathrm{ILCR}}_{{\text{Soilingestion }}} = \frac{{{\mathrm{TEQs}} \times \left( { {\mathrm{CSF}}_{{{\mathrm{ingestion}}}} \times \sqrt[3]{{\frac{{{\mathrm{BW}}}}{70} }} } \right) \times {\mathrm{IR}}_{{{\mathrm{soil}}}} \times {\mathrm{EF}} \times {\mathrm{ED}}}}{{{\mathrm{BW}} \times {\mathrm{AT}} \times 10^{6} }}$$

3 $${\text{ ILCR}}_{{\text{Soildermal }}} = \frac{{{\text{TEQs }} \times \left( { {\mathrm{CSF}}_{{{\mathrm{dermal}}}} { }x \sqrt[3]{{\frac{{{\mathrm{BW}}}}{70} }} } \right) \times {\text{SA }} \times {\mathrm{AF}} \times {\mathrm{ABS}} \times {\mathrm{EF}} \times {\mathrm{ED}}}}{{{\mathrm{BW}} \times {\mathrm{AT}} \times 10^{6} }}$$

4 $${\mathrm{ILCR}}_{{{\mathrm{Soilinhalation}}}} = \frac{{{\text{TEQs }} \times \left( {{\mathrm{CSF}}_{{{\mathrm{inhalation}}}} \times \sqrt[3]{{\frac{{{\mathrm{BW}}}}{70} }}} \right) \times {\mathrm{IR}}_{{{\mathrm{air}}}} \times {\mathrm{EF}} \times {\mathrm{ED}}}}{{{\mathrm{BW}} \times {\mathrm{AT}} \times {\mathrm{PEF}}}}$$

The total soil-related cancer risk was calculated as the sum of all routes:

5 $${\mathrm{ILCR}}_{{{\mathrm{Soil}}}} = {\mathrm{ILCR}}_{{{\mathrm{Soilingestion}}}} + {\mathrm{ILCR}}_{{{\mathrm{Soildermal}}}} + {\mathrm{ILCR}}_{{{\mathrm{Soilinhalation}}}}$$

## Results and discussion

### Concentration and spatial distribution of PAHs

Sixteen types of PAHs prioritized by the US EPA were detected in the surface soil samples (n = 42). The correlation coefficients of the calibration curves for the quantified PAH compounds ranged from 0.9922 (IcP) to 0.9995 (Ace). The limits of quantification (LOQ) varied between 0.05 ng/mL (BaP) and 7.04 ng/mL (Flu), while the method detection limits (MDL) were determined to be between 0.01 ng/g (BaP) and 2.12 ng/g (Flu). Recovery experiments indicated that percent recoveries ranged from 81.3 ± 11.9% for NaP to 99.7 ± 0.3% for Flu, all of which were within acceptable limits; therefore, measured PAH concentrations were not corrected for recovery. The relative standard deviation (RSD) values ranged from 0.35 (Flu) to 14.6 (NaP) (Table 2). As described in Sect. Quality assurance and quality control, the use of a representative composite matrix and blank signal subtraction effectively minimized matrix effects and improved analytical accuracy. Taken together, the applied matrix correction strategy, combined with acceptable recovery, %RSD, and compound-specific MDL/LOQ values, confirms that the analytical protocol used in this study yields reliable, accurate, and scientifically robust PAH concentration data (Table 2). Agreement with the certified values of NIST SRM 1647d further validates the accuracy of the method.

**Table 2:** Percent recoveries, the relative standard deviations, method detection limit, linearity and limit of quantification for the16 PAH compounds

Compound	%Recovery	%RSD	MDL ng/g	LOQ ng/mL	R^2^
NaP	81.3 ± 11.9	14.6	0.87	2.9	0.9991
Ace	94.4 ± 6.3	6.69	1.1	3.68	0.9995
Acy	94 ± 8.5	9.03	0.29	0.95	0.9993
Flr	93.4 ± 9.4	10.02	0.33	1.1	0.9989
Phe	90.1 ± 5.1	5.7	0.59	1.98	0.9989
Ant	98.0 ± 2.8	2.89	1.97	6.56	0.9966
Flu	99.7 ± 0.3	0.35	2.12	7.04	0.9936
Py	98.3 ± 2.9	2.94	0.42	1.4	0.9962
BaA	96.0 ± 5.7	5.89	1.4	4.6	0.9989
Chr	94.0 ± 8.5	9.03	0.03	0.1	0.999
BbF	97.0 ± 4.2	4.37	0.76	2.52	0.9991
BkF	98.5 ± 2.1	2.15	0.06	0.23	0.999
BaP	99.0 ± 1.4	1.43	0.01	0.05	0.9985
DahA	95.0 ± 7.1	7.44	0.06	0.21	0.999
BghiP	92.5 ± 10.6	11.47	0.04	0.15	0.999
IcP	95.0 ± 7.1	7.44	0.01	0.04	0.9922

The Kruskal–Wallis test was applied to evaluate differences among individual PAH concentrations within the summer dataset. The results indicated a statistically significant difference among the medians of the 16 PAHs (*p* < 0.05), suggesting heterogeneous distribution patterns of PAHs during the summer season. Similarly, the Kruskal–Wallis test applied to the winter dataset revealed statistically significant differences among the medians of the 16 PAHs (*p* < 0.05). Seasonal differences in total PAH concentrations (Σ16 PAHs) were evaluated using the two-sample Kolmogorov–Smirnov test. For each sampling site, individual PAH concentrations were summed to obtain total PAH values for summer and winter datasets. The Kolmogorov–Smirnov test revealed a statistically significant difference between the summer and winter distributions (D = 0.59, *p* < 0.05), indicating a pronounced seasonal variation in total PAH levels. The observed seasonal difference in total PAH concentrations suggests that variations in meteorological conditions, emission sources, and atmospheric processes play a significant role. Higher wintertime PAH levels may be attributed to increased combustion-related emissions and limited atmospheric dispersion.

For each individual PAH compound, the mean, median, standard deviation, minimum and maximum values, as well as the detection frequency for both summer and winter sampling campaigns, are presented in Table 3. In both seasons, IcP was the most frequently detected compound. In the summer samples, the least frequently detected compounds were Ace and Chr, whereas in the winter samples, Acy showed the lowest detection frequency. Examination of ΣPAH_16_ concentrations (ng/g dw) across the sampling sites revealed that the highest levels were associated with IcP in both seasons, with total concentrations of 6314.2 ng/g dw in summer and 13,687.7 ng/g dw in winter. Surface soil samples collected from 21 locations during both summer and winter exhibited total PAH concentrations ranging from 108.3 to 2587.8 ng/g dw in summer and from 111.8 to 3125.8 ng/g dw in winter. In order to provide a more systematic evaluation of soil contamination, Maliszewska-Kordybach (1996) introduced a four-tier classification scheme for PAH-polluted soils. According to this framework, soils with ΣPAH concentrations below 0.2 mg/kg are considered uncontaminated, while those with ΣPAH values between 0.2 and 0.6 mg/kg fall into the weakly contaminated category. ΣPAH concentrations ranging from 0.6 to 1.0 mg/kg are regarded as contaminated, whereas soils containing ΣPAH levels exceeding 1.0 mg/kg are classified as heavily contaminated. According to this contamination classification, 19.0% of the summer samples and 14.3% of the winter samples were classified as uncontaminated; 61.9% of the summer samples and 33.0% of the winter samples as weakly contaminated; 14.3% of both summer and winter samples as contaminated; and 4.7% of the summer samples and 33.3% of the winter samples as heavily contaminated.

**Table 3:** Istatistical parameters of individual PAHs for both sampling periods

Summer	NaP	Ace	Acy	Flr	Phe	Ant	Flu	Py
ΣPAH_16_ ng/g dw	7.0	14.0	15.6	75.1	94.7	77.2	364.9	159.7
Average	0.4	2.8	1.9	4.4	4.5	6.4	19.2	20.0
Median	0.3	0.4	0.6	1.0	4.4	2.2	11.9	4.5
Standard deviation	0.2	4.5	3.4	12.9	1.8	13.9	25.4	41.1
Minimum	0.2	0.2	0.2	0.4	1.0	0.7	2.0	1.1
Maximum	0.8	10.7	10.2	54.5	8.4	50.2	96.2	121.0
Frequency	**19**	**5**	**8**	**17**	**21**	**12**	**19**	**8**

Summer	BaA	Chr	BbF	BkF	BaP	DahA	BghiP	IcP
ΣPAH_16_ ng/g dw	27.5	76.3	60.5	101.1	107.6	67.2	30.3	6314.2
Average	4.6	15.3	8.6	11.2	6.7	7.5	5.1	315.7
Median	1.3	15.2	1.1	2.5	4.2	3.6	2.8	166.4
Standard deviation	5.7	17.5	12.3	14.2	7.0	7.1	5.9	387.1
Minimum	0.7	0.6	0.5	1.2	1.6	1.1	0.3	78.6
Maximum	14.2	43.5	29.8	39.7	27.6	17.3	14.8	1789.6
Frequency	**6**	**5**	**7**	**9**	**16**	**9**	**6**	**20**

Winter	NaP	Ace	Acy	Flr	Phe	Ant	Flu	Py
ΣPAH_16_ ng/g dw	26.6	72.1	1.7	105.7	140.4	142.4	877.8	367.2
Average	1.3	12.0	1.7	6.6	15.6	23.7	67.5	36.7
Median	0.6	9.6	1.7	3.6	12.0	7.8	41.4	14.4
Standard deviation	1.7	12.4		7.7	10.5	36.0	87.6	64.7
Minimum	0.3	0.6	1.7	1.4	4.6	1.3	2.7	5.5
Maximum	7.6	33.8	1.7	30.0	33.0	95.4	336.5	216.7
Frequency	**20**	**6**	**1**	**16**	**9**	**6**	**13**	**10**

Winter	BaA	Chr	BbF	BkF	BaP	DahA	BghiP	IcP
ΣPAH_16_ ng/g dw	145.3	52.0	73.1	119.3	117.0	83.5	70.8	13,687.7
Average	18.2	6.5	7.3	9.2	6.9	6.4	5.1	651.8
Median	11.4	5.4	6.7	7.3	6.4	6.8	4.0	415.8
Standard deviation	21.0	4.9	4.2	6.1	3.7	1.5	5.4	733.6
Minimum	1.0	1.1	2.1	3.0	1.5	1.3	0.7	59.2
Maximum	65.5	13.9	17.2	24.4	17.6	6.9	22.2	3099.2
Frequency	**8**	**8**	**10**	**13**	**17**	**13**	**14**	**21**

When total PAH concentrations are categorized by ring number (with 2–3-ring PAHs comprising NaP, Ace, Acy, Flr, Phe, and Ant; 4-ring PAHs comprising Flu, Py, BaA, and Chr; 5-ring PAHs comprising BbF, BkF, BaP, and DahA; and 6-ring PAHs comprising BghiP and Icp), it becomes evident that the winter sampling results are markedly higher than those obtained during the summer sampling period (Table 4).

**Table 4:** Total PAH concentrations (ng/g dw) in summer and winter sampling according to ring number

ΣPAH_16_ ng/g dw	2–3 ringed	4 ringed	5 ringed	6 ringed
Summer	283,5	628,5	336,5	6344,5
Winter	489,0	1442,3	392,9	137585

During the summer, recreational activities, particularly picnics with barbecues, reached their highest levels, whereas an increase was observed in winter due to different factors. The summer sampling was conducted in June, when the region’s geographic location results in relatively mild temperatures and recreational activities were just beginning. Visitor numbers typically increase further in July, August, and September as temperatures rise. Local authorities’ visitor statistics were collected where available; for instance, in 2010, 177,519 people visited according to the Ministry of Forestry and Water Affairs, IX Regional Directorate, while in 2015, Bolu Governorship reported 88,316 vehicles and 319,461 visitors to Gölcük Nature Park (Bolu Governorship, 2017; Ministry of Forestry & Water Affairs, 2017). The relatively high PAH levels observed in the winter sampling can be partly attributed to the fact that recreational activities had not fully started during the June summer sampling. Although direct PAH measurements are not available for the peak activity months (July–September), the increasing visitor numbers during this period support the inference that cumulative PAH deposition from these more intense activities likely contributed to the elevated winter concentrations. Consequently, cumulative PAH deposition resulting from the more intense activities in July, August, September, and to some extent October, likely contributed to the elevated concentrations measured in winter. As reported in the literature, some PAHs are resistant to biodegradation and prone to bioaccumulation, and although they are present in all environmental compartments, they preferentially settle onto the upper soil layer through dry and wet deposition, forming an important accumulation reservoir due to their strong affinity for soil organic matter (Aichner et al., 2015; Liu et al., 2014; Melnyk et al., 2015; Nadal et al., 2004; Sweetman et al., 2005).

Another major factor is the photo-oxidation of PAH compounds. PAHs in soils are known to be strongly influenced by temperature and solar radiation (Balmer et al., 2000; Frank et al., 2002; Gong et al., 2001; Xiaozhen et al., 2005), which can also explain the relatively low concentrations observed in June. Low-molecular-weight PAHs can be transported over longer distances from their sources through atmospheric processes due to their higher vapor pressures; in addition, the volatility of these compounds should not be overlooked (Mallick et al., 2011; Tanic et al., 2023).

Meteorological data for 01 May–30 June 2016 and 01 October–30 November 2016 (Fig. S1 and S2) indicate that average temperatures ranged from 10–25 °C in summer and 0–16 °C in autumn, with rainfall being generally low in summer and higher in autumn. ΣPAH₁₆ concentrations in soil ranged from 7.0 to 6314.2 ng/g in summer and 1.7 to 13,687.7 ng/g in autumn. Low molecular weight PAHs (NaP, Ace, Acy) generally exhibited low concentrations in summer, whereas high molecular weight PAHs (BkF, BaP, DahA, BghiP, IcP) showed elevated levels in certain samples. The combination of high temperatures and limited rainfall in summer favored PAH accumulation in soils, while limited wash-off restricted the transport of low molecular weight PAHs. In autumn, increased rainfall promoted partial transport of low molecular weight PAHs, whereas high molecular weight PAHs remained persistent due to limited degradation under cooler conditions. Analysis of PAH concentrations in relation to seasonal rainfall indicates that meteorological conditions strongly influence both the persistence and mobility of PAHs, with clear differences between low and high molecular weight compounds across seasons.

Pearson correlations of individual PAH compounds were examined for the soil samples collected during the summer and winter sampling periods. In the summer dataset, some PAH compounds could not be included in the analysis due to their low detection frequency. In the summer samples, Flr exhibited statistically significant correlations with NaP, Phe, Ant, BaP, and IcP; Ant showed significant correlations with Phe, BaP, and IcP; and BkF was significantly correlated with Py, BbF, BaP, and IcP at *p* < 0.05 and the 95% confidence level (Table 5). In the winter samples, NaP showed statistically significant correlations with Acy, Phe, and Flu; Acy correlated significantly with Flr, Ant, Chr, BaP, and IcP; Ace with BaA, DahA, and BghiP; Phe with BaP; Ant with Chr and BaP; Chr with BaP; and BbF with BkF, all at *p* < 0.05 and the 95% confidence level (Table 6).

**Table 5:** Pearson correlations for the summer samples

**Summer**	NaP	Ace	Flr	Phe	Ant	Flu	Py	BbF	BkF	BaP	IcP
NaP											
Ace	0,00										
Flr	**0,56**	− 0,17									
Phe	− 0,04	0,49	**0,56**								
Ant	0,22	0,27	**0,99**	**− 0,60**							
Flu	− 0,15	− 0,07	− 0,10	0,05	− 0,06						
Py	0,18	− 0,33	− 0,23	− 0,06	0,17	− 0,19					
BbF	− 0,20	− 0,33	− 0,19	− 0,05	− 0,24	0,40	0,46				
BkF	0,01	− 0,35	− 0,19	− 0,26	− 0,20	0,12	**0,69**	**0,78**			
BaP	0,45	− 0,18	**0,76**	− 0,43	**0,72**	− 0,05	0,12	0,32	− 0,01		
IcP	− 0,04	− 0,17	**0,89**	− 0,36	**0,95**	0,05	− 0,26	− 0,17	0,20	**0,60**	

Bold values indicate pairs with statistically significant correlations

**Table 6:** Pearson correlations for the winter samples

	NaP	Acy	Ace	Flr	Phe	Ant	Flu	Py	BaA	Chr	BbF	BkF	BaP	DahA	BghiP	IcP
NaP																
Acy	**0,88**															
Ace	− 0,04	0,17														
Flr	0,26	**0,94**	− 0,16													
Phe	**0,79**	− 0,26	0,05	− 0,04												
Ant	0,12	**0,99**	− 0,23	0,30	− 0,20											
Flu	**0,85 **	− 0,09	− 0,06	0,16	0,65	0,02										
Py	− 0,14	− 0,15	− 0,21	− 0,20	− 0,18	− 0,14	− 0,16									
BaA	0,04	− 0,03	**0,98**	− 0,23	− 0,20	− 0,02	− 0,18	0,08								
Chr	− 0,36	**0,89**	0,04	0,78	− 0,45	**0,93**	− 0,34	0,17	0,06							
BbF	− 0,19	− 0,19	− 0,31	− 0,18	− 0,17	− 0,03	0,02	0,38	− 0,23	0,23						
BkF	− 0,08	− 0,41	− 0,37	− 0,17	− 0,09	0,04	− 0,09	0,66	0,01	0,11	**0,67**					
BaP	0,06	**0,78**	− 0,17	**0,57**	− 0,23	**0,58**	0,03	− 0,04	− 0,15	**0,73**	0,38	0,12				
DahA	− 0,15	− 0,32	**− 0,93**	− 0,22	− 0,15	− 0,32	− 0,06	− 0,21	− 0,29	− 0,39	− 0,23	− 0,18	0,02			
BghiP	0,12	− 0,41	**0,83**	− 0,27	0,21	− 0,44	0,00	− 0,02	0,34	− 0,30	0,10	0,53	− 0,25	− 0,06		
IcP	0,09	**0,89**	− 0,17	0,41	0,20	0,53	0,13	− 0,14	− 0,19	0,48	− 0,04	− 0,13	0,37	− 0,12	0,03	

Bold values indicate pairs with statistically significant correlations

A detailed evaluation of the sampling points indicates that their spatial positioning within the nature park plays a critical role in the interpretation of PAH distribution patterns. Beginning at the park entrance, the eastern sector constitutes the only designated lakeside area where barbecuing is permitted; this zone includes sampling points 1, 2, 3, 4, 5, 6, 7, 19, 20, and 21, and is classified as the eastern lakeside picnic barbecue area. This area, along with the northern lakeside, is occasionally exposed to direct sunlight despite being primarily considered non-shaded. Additional sampling regions include the northern lakeside (points 8, 9, 10, 11, and 12), the western shaded lakeside (point 13), the southern shaded lakeside (points 14, 15, and 16), and the south-eastern lakeside adjacent to the barbecue area (points 17 and 18). As previously noted, PAH concentrations are governed by several environmental drivers, particularly photo-oxidation processes and seasonal temperature variations. Consequently, site-specific characteristics—such as exposure to direct sunlight versus shading—are expected to influence PAH accumulation and degradation dynamics. Table 7 presents the mean concentrations of PAHs (ng/g dw), grouped by ring number and categorized according to sampling-site characteristics and season.

**Table 7:** Mean concentrations of PAHs (ng/g dw) by ring number at different lakeside sampling locations and seasons

Average ng/g dw	2–3 ringed	4 ringed	5 ringed	6 ringed
Eastern lakeside picnic–barbecue area-summer	176.1	70.9	34.3	333.1
Eastern lakeside picnic–barbecue area-winter	52.0	96.5	29.7	297.1
Northern lakeside-summer	9.0	37.5	29.9	402.4
Northern lakeside-winter	23.6	441.9	29.1	747.2
Western shaded lakeside-summer	2.4	n.d.*	6.1	269.5
Western shaded lakeside-winter	24.4	348.2	18.1	1444.9
Southern shaded lakeside-summer	8.4	514.3	3.7	130.5
Southern shaded lakeside-winter	91.0	703.1	20.6	821.5
Southeastern lakeside, adjacent to the barbecue area-summer	9.2	155.3	41.1	187.4
Southeastern lakeside, adjacent to the barbecue area-winter	12.4	19.5	27.3	1579.2

^*^ not detected

Analysis of the 2–3-ring PAH group revealed that winter concentrations exceeded summer concentrations across all sampling regions except the southern shaded lakeside. A similar pattern was observed for 4-ring PAHs, with winter concentrations surpassing summer values at all locations except the south-eastern lakeside adjacent to the barbecue area. These trends are largely attributed to the substantial increase in recreational activity—and thus potential PAH inputs—from July through October, following the June sampling campaign during which recreational pressure had only recently begun due to climatic conditions. The cumulative deposition resulting from these late-summer and early-autumn activities appears to contribute significantly to elevated winter concentrations. Site-specific deviations from the general seasonal trends, such as the lower winter concentrations observed at the southern shaded lakeside and the distinct seasonal pattern at the south-eastern lakeside adjacent to the barbecue area, are likely influenced by microenvironmental factors, including shading, intermittent exposure to direct sunlight, and localized recreational activities. These considerations are integral to the interpretation of seasonal variations in PAH concentrations across the different sampling locations. Despite this accumulation, the southern shaded lakeside exhibited lower PAH levels in winter compared to summer. This deviation is likely associated with intermittent exposure to intense solar radiation, which may have promoted enhanced photo-oxidation of PAHs even in an otherwise shaded microenvironment When the mean summer and winter concentrations of 5-ring PAHs were examined across the sampling regions, the values were found to be either nearly identical or only slightly higher in one season compared to the other. Therefore, no meaningful difference in the average concentrations of 5-ring PAHs between summer and winter sampling campaigns could be identified based on sampling location. Similar to the 2–3-ring PAH compounds, the 6-ring PAH compounds exhibited higher concentrations in the summer only at the sampling points located in the eastern lakeside picnic–barbecue area. The Northern lakeside and the Eastern lakeside picnic–barbecue area are occasionally exposed to direct sunlight, which may influence the photodegradation of PAHs in these otherwise non-shaded sites and should be considered when interpreting seasonal concentration differences. In all other regions, however, winter concentrations were higher than summer concentrations, which can be attributed to the same factors described above. When interpreting these results, it should be noted that undesired variations in concentrations may arise from factors such as the number of visitors, dust resuspended from walking or other activities, coal dust and/or ash dispersed during barbecue coal handling, and direct fat droplets dripping onto the ground from cooked meat.

To assess the pollution level of Gölcük Nature Park, both a comparison with the existing literature was performed and as described in the Materials and Methods section, three surface soil samples were collected from the shoreline of a reference lake and analyzed for PAHs under the same conditions. The mean total PAH concentration for the reference lake was 140.4 ng/g dw, while the mean total PAH concentrations for the summer and winter sampling periods in Gölcük Nature Park were 361.6 ng/g dw and 765.8 ng/g dw, respectively. Based on the PAH concentration levels detected in the surface soils of the selected reference lake, it is evident that the surface soil samples from Gölcük Nature Park exhibit substantially higher concentrations. A review of similar studies in the literature indicates that, although no research has been identified in a natural setting such as a nature park, as conducted in the present study, numerous investigations have been carried out in urban recreational areas, particularly in children’s playgrounds, to determine the concentrations of the 16 priority PAH compounds in surface soils. or instance, in a study conducted in Stockholm city center between 2016 and 2017, a total of 374 surface soil samples (10–20 cm) collected from 25 different parks exhibited Σ16PAH concentrations ranging from 8.0 to 12.2 mg/kg (Dreij et al., 2020). In Seville, Spain, Σ15PAH concentrations measured in 41 surface soil samples (0–10 cm) ranged from 0.09 to 4.0 mg/kg (Morillo et al., 2008). In Krakow and Zakopane, Poland, ΣPAH concentrations determined in 19 surface soil samples (0–10 cm) collected from residential and park areas varied between 55 and 1180 mg/kg (Ciarkowska et al., 2019). As shown in Table 8, the PAH concentration levels detected in the nature park surface soils in the present study are considerably higher compared to those reported for urban park soils in the literature. The high concentrations can be attributed to the fact that the study area is densely surrounded by trees and lacks a dominant wind flow, causing barbecue smoke generated within the park to remain trapped over the area. Over the years, this smoke has gradually deposited onto the surface soils, resulting in the accumulation of PAHs and ultimately leading to elevated concentration levels.

**Table 8:** Summary statistics for total PAH concentration in urban soils in different cities

Sampling period	Median mg/kg	Mean mg/kg	Range mg/kg	Compounds	Depth	Sample size	Sampling area	References
2016	387	684	108–3125	PAH_16_	0–10 cm	n = 42	Bolu, Türkiye	This study
1994–2007	0.24	–	0.01–18.0	PAH_16_	0–2 cm	n = 75	Trondheim Norway	Ottesen et al., (2008)
1994–2007	2.05		0.03–81	PAH_16_	4–5 cm	n = 435	Bergen, Norway	Ottesen et al., (2008)
2007–2008	–	2.7	0.06–73.3	PAH_16_	–	n = 100	Lisbon, Portugal	Marinho Reis et al., (2016)
2012	–	2.1	0.4–12.1	PAH_16_	0–10 cm	n = 61	Bratislava, Slovakia	Hiller et el., (2015)
2014	–	4.8		PAH_16_	10-20 cm	n = 374	Stocholm, Sweden	Dreij et al., (2020)
2016	–	0.46	0.07–6.68	PAH_16_	0–10 cm	n = 122	Urban parks in Beijing, China	Qu et al., (2020)
	–	–	0,1–3,2	PAH_15_	0–10 cm	n = 41	Sevilla Spain	Morillo et al., (2008)
2016	–	0.45	0.3–0.5	PAH_16_	0–10 cm	–	Krakow, Poland	Ciarkowska et al., (2019)

### PAH molecular composition analysis

**Fig. 2:**
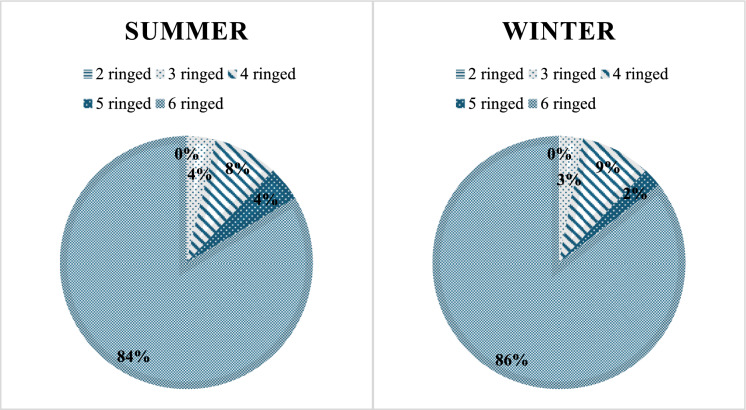
Contribution of PAHs to total concentrations in summer and winter samples by number of aromatic rings

When classified by the number of aromatic rings, PAHs in the soils of the nature park showed that 2–3 ring compounds contributed 4% of the total PAH concentration in summer and 3% in winter. The low contribution of 2–3 ring PAHs in soils can be explained by their reduced persistence in the soil environment; contributing factors include their relatively low sorption to soil matrices (Ter laak et al., 2006), higher volatility (Park et al., 1990), and greater susceptibility to biological degradation (Oleszczuk & Baran, 2003). 4-ring PAHs accounted for 8% and 9% of the total PAHs in summer and winter, respectively, while 5-ring PAHs contributed 4% in summer and 2% in winter. In contrast, 6-ring PAHs represented the largest proportion of total PAH concentrations (84% in summer and 86% in winter) (Fig. 2). The substantial accumulation of 6-ring PAHs can be attributed to their physicochemical properties: their low water solubility limits removal and dilution by rainwater and runoff, and their high boiling points prevent significant volatilization, even under elevated summer temperatures. Furthermore, these compounds are more stable than low molecular weight PAHs and are considerably less susceptible to photo oxidation compared with 2–3 ring PAHs. The dominance of high molecular weight PAHs over low molecular weight PAHs is a well-documented characteristic of urban soils in various cities; in many studies, the mean contribution of HMW PAHs to Σ16PAHs exceeds 80% (e.g., Cachada et al., 2012; Liang et al., 2011; Liu et al., 2010).

That the compositional patterns of PAHs serve as useful indicators for identifying pollution sources was demonstrated by Qu et al. (2020). In general, 2-ring PAH are predominantly associated with oil spill events, while 3- and 4-ring PAHs are mainly generated from the combustion of coal and biomass. Gasoline-fueled engines tend to emit higher proportions of 5-ring PAHs, whereas diesel combustion is characterized by the formation of 6-ring PAHs (Wang et al., 2018; Yu & Shao, 2007). Considering individual compounds, Nap can also arise from incomplete combustion processes (Simcik et al., 1999). Acy has been identified as a distinctive marker of fuel-wood combustion (Biache et al., 2014). BkF, Chr, and Pyr are largely generated through industrial coal combustion (Brown & Brown, 2012). Moreover, automobile exhaust represents the principal source of IcP, BghiP, and DahA (Hong et al., 2007). It should also be noted that the substantial contribution of 6-ring PAH compounds is significantly influenced by emissions from diesel-fueled buses used for organized tours to the nature park, as evidenced by visitor numbers. PAH composition by ring number was calculated using aggregated data for the entire park to provide a holistic overview of spatial trends. While site-specific factors such as barbecue activity or shading influence local concentrations and are addressed in other sections, this general analysis offers a park-wide perspective on PAH distribution. Therefore, interpretations at individual locations should be considered in the context of this broader, aggregated assessment. An initial evaluation of the contributions of individual PAH compounds in the study area shows that soils contain substantially higher levels of 4–6 ring PAHs compared with 2–3 ring PAHs, indicating that the PAHs present in the nature park mainly derive from high-temperature combustion of fossil fuels such as coal, gasoline, and diesel. While these compositional patterns provide valuable insights into potential combustion sources, it should be noted that the dominance of 5–6 ring PAHs cannot be unambiguously attributed to barbecuing alone, as similar distributions may arise from other high-temperature combustion sources. Consequently, further source-specific interpretations should be considered indicative rather than definitive, particularly given the limited literature on PAH emissions specifically originating from barbecue activities.

### Diagnostic ratios

**Fig. 3:**
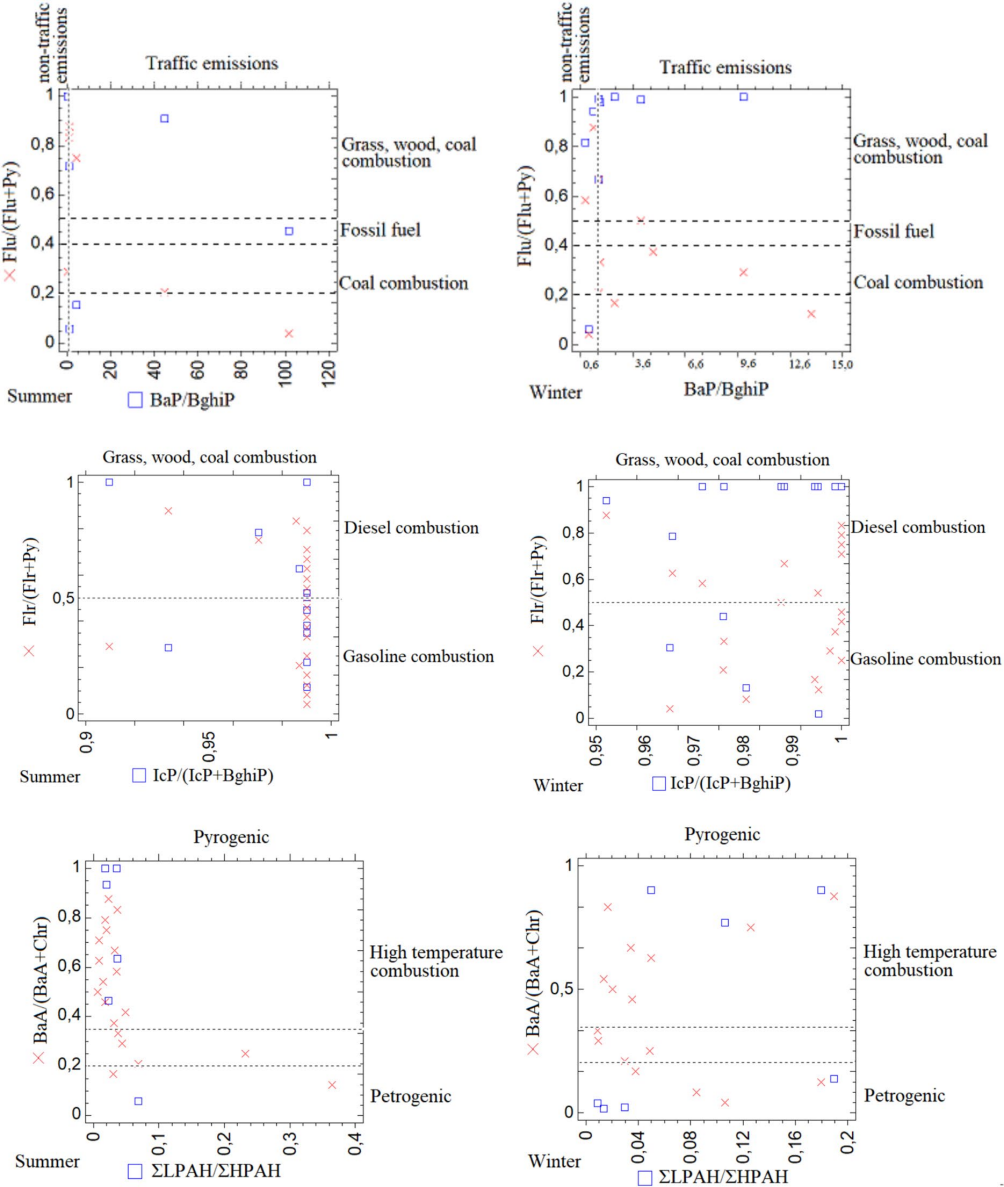
Diagnostic ratio charts

Diagnostic ratios (Table 1) were used to identify the potential source profiles of PAHs. Based on the BaA/(BaA+Chr) ratio, 71.5% of the summer samples and 61.9% of the winter samples were classified as originating from high-temperature combustion, while the remaining samples were attributed to petrogenic sources. Furthermore, the ratio of total LPAHs to total HPAHs indicated that all samples, in both summer and winter sampling periods, were derived from pyrogenic sources. Pyrogenic high-molecular-weight PAHs, consisting of four to six rings and originating primarily from the incomplete combustion of organic materials such as fossil fuels, vehicle emissions, and waste-tire or biomass burning, contrast with petrogenic low-molecular-weight PAHs, which contain two to three rings and are associated with coal or petroleum (Jang et al., 2013; Sany et al., 2014; Santos et al., 2017; Bielińska et al., 2018). Based on the Flr/(Flr+Py) ratios, the potential source profiles for both summer and winter samples were almost evenly divided between gasoline and diesel combustion, indicating a strong influence from the types of fuels used by vehicles driven by visitors to the area. According to the IcP/(IcP+BghiP) ratios, all samples from both seasons were attributed exclusively to grass, wood, and coal combustion, clearly reflecting the impact of intensive barbecuing activities in the park. The BaP/BghiP ratios further showed that more than 85% of the samples in both seasons originated from non-traffic emissions. Similarly, the Flu/(Flu+Py) ratios indicated that the likely source for both sample sets was grass, wood, and coal combustion, consistent with the IcP/(IcP+BghiP) diagnostic ratio. (Fig. 3). The calculated diagnostic ratios were evaluated for each sample and season, and the relative contributions of different PAH sources were expressed as percentage distributions based on the frequency of source-indicative ratio ranges.

### Seasonal mapping of PAH concentrations

**Fig. 4:**
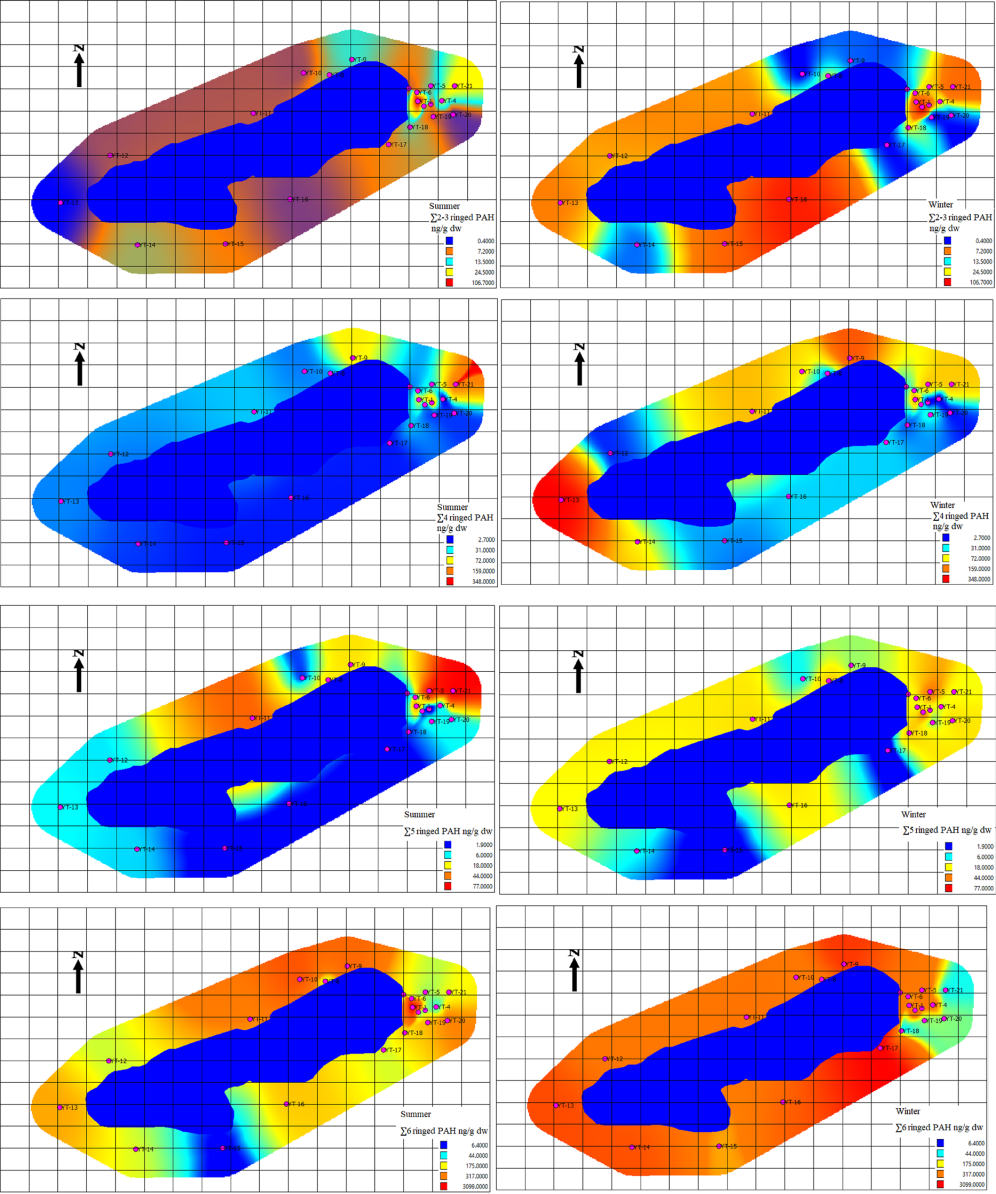

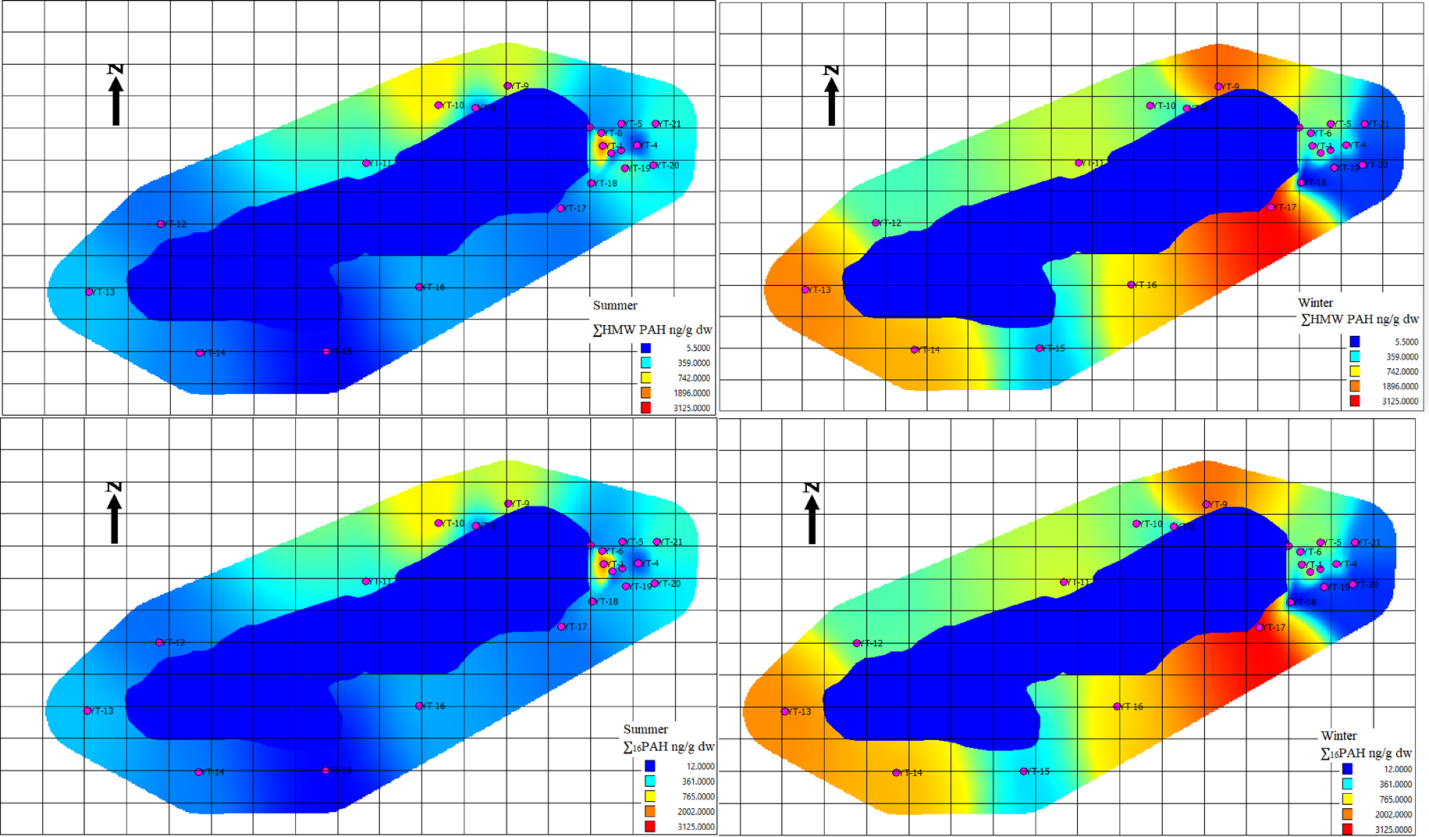
Seasonal mapping of PAH concentrations

To visualize the spatial dynamics of PAH accumulation in Gölcük Nature Park, seasonal distribution maps of ΣPAH_16_ and PAH subgroups (2–3 ring, 4 ring, 5 ring) were generated for both summer and winter sampling periods. These IDW-based maps are intended primarily to provide a visual and qualitative comparison of seasonal PAH distribution, rather than to serve as exact quantitative predictions. When creating the maps, each PAH group (2–3 ring, 4 ring, 5 ring, total) was plotted using the same color scale for summer and winter to allow direct seasonal comparisons, and the maps were arranged side by side; this approach facilitates a clearer visual interpretation of spatial distribution changes across seasons. During the summer, elevated ΣPAH_16_ values were predominantly concentrated in the eastern lakeside picnic–barbecue areas, reflecting recent emissions from recreational grilling activities. In contrast, the winter maps exhibited a more widespread and intensified contamination pattern, with high concentrations extending across the northern and western lakeside areas. During winter, high-molecular-weight PAHs (5-ring) were particularly persistent in these high-concentration zones, with degradation being more limited compared to summer; this observation suggests that lower temperatures and reduced microbial activity enhance the persistence of high-molecular-weight PAHs. The expansion of high-concentration zones is consistent with the cumulative deposition of PAHs from extensive late-summer and early-autumn recreational activities combined with the limited degradation of high-molecular-weight PAHs during colder months. The spatial heterogeneity observed in both seasons highlights the influence of microenvironmental factors—such as shading, wind confinement due to dense forest cover, and differential sunlight exposure—on PAH persistence and degradation. Additionally, topographic variations and differences in shoreline vegetation density should be considered, as they may create localized differences in PAH accumulation and distribution. Overall, the seasonal maps indicate that winter soils retain substantially higher PAH loads, suggesting that deposition exceeds degradation following peak recreational activity periods (Fig. 4).

### Risk assessment of PAHs

For the calculation of toxicity equivalent concentration (TEQs) values, the median BaP concentrations from the summer and winter samples were used.

Soil-based ILCR values via ingestion, dermal contact, and inhalation for different age and sex groups are presented in Table 9 for summer and winter. Overall, ILCR values were higher in winter compared to summer across all age groups, reflecting a seasonal increase in potential cancer risk. This seasonal variation may be attributed to enhanced PAH accumulation in soils during colder months, decreased degradation, and limited dispersion. Age-related differences were also observed. Adults generally exhibited higher ILCR values than children and adolescents, with dermal contact contributing most significantly to total risk. For instance, in winter, ILCRsoil, ILCRdermal values for adult males and females were 0.003329 and 0.003605, respectively. These differences among age groups are largely due to parameters used in the ILCR calculations, such as body weight, exposure duration, soil ingestion rate, skin surface area, and other coefficients derived from the literature. Minor sex-related differences were noted as well, with adult females exhibiting slightly higher ILCR values than males, likely reflecting variations in body weight, skin surface area, and behavioral exposure patterns. According to the USEPA and related regulatory frameworks, an ILCR of 10^−6^ or below is considered an acceptable or negligible risk, whereas values equal to or exceeding 10^−4^ indicate a significant health concern requiring intervention. Values between 10^−6^ and 10^−4^ represent a potential health risk (USEPA, 2011). USEPA further recommends a target ILCR of ≤ 10^−6^, or as low as reasonably practicable (ALARP), achievable through source reduction, engineering controls, or administrative measures, followed by recalculation to ensure compliance (USEPA, 2011). In this study, ILCR values calculated for soil exposure via ingestion, dermal contact, and inhalation indicated elevated potential health risks across all age and sex groups, particularly during winter months. Total ILCR values ranged from 0.002625 to 0.0040418 in summer and 0.004054 to 0.006242 in winter, exceeding the USEPA’s acceptable risk threshold (10^−4^). Dermal contact and soil ingestion were the dominant exposure pathways, while inhalation contributed negligibly (< 10^−7^). These findings highlight seasonal and demographic variations in risk, with adults—especially females—experiencing the highest ILCR values, emphasizing the need for effective risk management strategies to reduce human exposure to PAH-contaminated soils. To mitigate these risks, several practical management recommendations can be considered. For example, daily visitor limits or a reservation system could be implemented for activities such as barbecues and other recreational use of the area. In addition, periodic monitoring and assessment of soil PAH levels should be conducted to track changes over time. These measures can contribute to the reduction of potential health risks, particularly for children and other high-exposure groups, and are important for the effective management of environmental PAH exposure.

**Table 9:** ILCR summer and winter values of soils for childhood, adolescence and adulthood through ingestion, dermal contact, and inhalation

	Childhood		Adolescence		Adulthood	
	Male	Female	Male	Female	Male	Female
Summer						
ILCRsoilingestion	0,001091	0,0011223	0,00171719	0,0019579	0,001577	0,0017076
ILCRsoildermal	0,001534	0,0015779	0,00084498	0,00096342	0,002155	0,002334
ILCRsoilinhalation	1,86E−08	1,909E−08	1,1683E−07	1,332E−07	1,07E−07	1,162E−07
ILCRs	0,002625	0,0027003	0,00256229	0,00292146	0,003732	0,0040418
Winter						
ILCRsoilingestion	0,001685	0,0017333	0,00265197	0,00302371	0,002435	0,0026372
ILCRsoildermal	0,002369	0,0024369	0,00130495	0,00148788	0,003329	0,0036046
ILCRsoilinhalation	2,87E−08	2,948E−08	1,8042E−07	2,0571E−07	1,66E−07	1,794E−07
ILCRs	0,004054	0,0041702	0,00395711	0,00451179	0,005764	0,006242

## Conclusion

This study provides one of the first comprehensive assessments in Türkiye of barbecue-related PAH contamination in a protected recreational area. Surface soils in Gölcük Nature Park exhibited Σ_16_PAH levels exceeding those reported for many urban and industrial regions, demonstrating that intensive recreational activities constitute a significant and often underestimated PAH source. Seasonal analyses revealed pronounced winter accumulation driven by cumulative deposition from peak summer–autumn barbecuing, while the dominance of 6-ring PAHs reflected strong environmental persistence. Comparison with a reference lake further confirmed the elevated pollution load attributable to recreational pressure.

While park-soil assessments in the literature predominantly focus on hygiene and public-health risks, our findings highlight the necessity of evaluating the ecological condition of soils to ensure the long-term sustainability of green spaces. Such evaluations should rely on holistic approaches that integrate physical, chemical, and biological indicators and that remain applicable across diverse climatic and urban landscape settings. Overall, the results underscore the need for improved management of barbecue activities, systematic ecological monitoring, and policy measures aimed at protecting natural parks from long-term pollutant accumulation.

## Supplementary Information

Below is the link to the electronic supplementary material.

Supplementary file1 (DOCX 72 KB)

## Data Availability

The data used for this study are held by the authors. Please contact the author for details.
