# Supplementary material for: Seasonal dynamics of barbecue-derived PAH accumulation in recreational Nature Park soils: evidence from Bolu Gölcük, Türkiye
Source: Environ Geochem Health. 2026 Feb 9;48(3):152. doi: 10.1007/s10653-026-03047-5 (PMC12886246; doi:10.1007/s10653-026-03047-5)
Supplement: Supplementary file 1 — Supplementary file1 (DOCX 72 KB) [file 10653_2026_3047_MOESM1_ESM.docx]

**Seasonal Dynamics of Barbecue-Derived PAH Accumulation in Recreational Nature Park Soils: Evidence from Bolu Gölcük, Türkiye**

Melike Büşra Bayramoğlu Karşı^1^, Ercan Berberler^2,3^, Duran Karakaş^2^

*^1^ Innovative Food Technologies Development Application and Research Centre, YENIGIDAM, Bolu Abant Izzet Baysal University, 14030, Bolu, Türkiye*

*^2^ Department of Environmental Engineering Bolu Abant Izzet Baysal University, 14030, Bolu, Türkiye*

*^3^ Department of Environmental Engineering, Bartın University, 74100, Bartın, Türkiye*

*Corresponding author e-mail: [melikebayramoglu@ibu.edu.tr](mailto:melikebayramoglu@ibu.edu.tr)


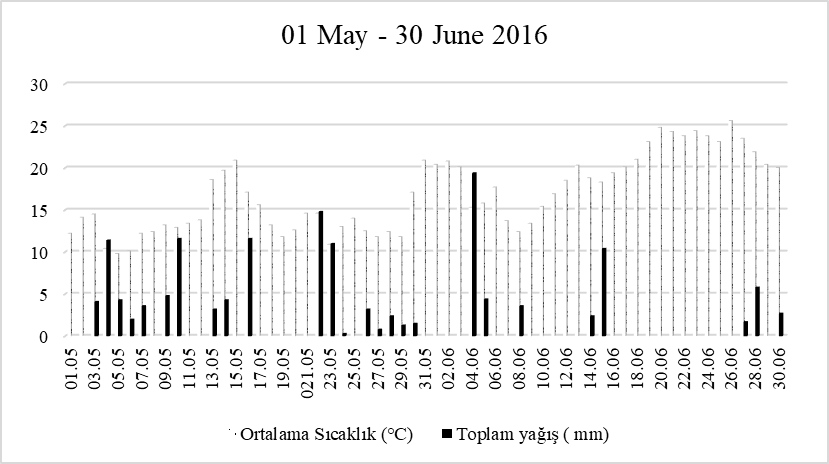

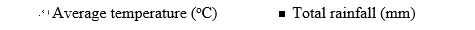


**Figure S1.**  01.05-30.06.2016 average temperature (^o^C) and total rainfall (mm)

**Figure S2.**  01.10-30.11.2016 average temperature (^o^C) and total rainfall (mm)

**Table S3.** Parameters for cancer risk of soil

| Definition | units | Childhood | | Adolescence | | Adulthood | | References |
| --- | --- | --- | --- | --- | --- | --- | --- | --- |
|  |  | Male | Female | Male | Female | Male | Female |  |
| CSF_ingestion_ | mg·d^-1^ kg^-1^ | 7,3 | | | | | | USEPA (1994) |
| CSF_Dermal_ | mg·d^-1^ kg^-1^ | 25 | | | | | | USEPA (1994) |
| CSF_Inhalation_ | mg·d^-1^ kg^-1^ | 3,85 | | | | | | USEPA (1994) |
|  | | **Childhood** | | **Adolescence** | | **Adulthood** | |  |
|  |  | Male | Female | Male | Female | Male | Female |  |
| Body weight (BW) | kg | 21,44 | 20,55 | 71,1 | 58,4 | 80,8 | 71,7 | TUIK (2022), CHSA (2006) |
| Exposure frequency (EF) | D year^-1^ | 350 | 350 | 350 | 350 | 350 | 350 | USEPA (1996) |
| Exposure duration (ED) | year | 2 | 2 | 7 | 7 | 7 | 7 | Peng et al (2011), USEPA (1996) |
| Inhalation rate (*IRair*) | m^3^ d^-1^ | 5 | 5 | 20 | 20 | 20 | 20 | Peng et al (2011), USEPA (1996) |
| Soil intake rate (*IRsoil*) | mg d^-1^ | 114 | 114 | 114 | 114 | 114 | 114 | USEPA (1996) |
| Dermal surface exposure (SA) | cm^2^ d^-1^ | 1800 | 1800 | 1800 | 1800 | 5000 | 5000 | Peng et al (2011) |
| Dermal adherence factor (AF) | mg cm^-2^ | 0,2 | 0,2 | 0,07 | 0,07 | 0,07 | 0,07 | USEPA (1996) |
| Dermal adsorption fraction (ABS) | unitless | 0,13 | 0,13 | 0,13 | 0,13 | 0,13 | 0,13 | USEPA (1996) |
| Average life span (AT) | year | 70 | 70 | 70 | 70 | 70 | 70 | Peng et al (2011) |
| Particulate emission factor (PEF) | m^3^ kg^-1^ | 1.36 × 10^9^ | 1.36 × 10^9^ | 1.36 × 10^9^ | 1.36 × 10^9^ | 1.36 × 10^9^ | 1.36 × 10^9^ | USEPA (1996) |

**References**

China Health Statistics Annual. National Health and family Planning Commission of peoples of China. 2006. <http://www.nhfpc.gov.cn/htmlfiles/zwgkzt/ptjnj/year2006/index.html>.

Peng, C., Chen, W., Liao, X. 2011. Polycyclic aromatic hydrocarbons in urban soils of Beijing: status, sources, distribution and potential risk. Environ. Pollut. 159(3), 802-808.

Turkish Statistical Institute (TUIK). (2022), Average Body Weights by Age Group and Gender, 2008–2022, https://data.tuik.gov.tr/Kategori/GetKategori?p=saglik-ve-sosyal-koruma-101&dil=1 Access date:28.04.2025.

USEPA, 1994. “Benzo[a]pyrene (BaP) (CASRN 50-32-8)”. USA. <http://www.epa.gov/ ncea/iris/subst/0136.htm>.

USEPA, 1996. Waste and Cleanup Risk Assessment. EPA/540/R95/128 May. Washington, DC, USA.
